# Protein Name Tagging Guidelines: Lessons Learned

**DOI:** 10.1002/cfg.452

**Published:** 2005

**Authors:** Inderjeet Mani, Zhangzhi Hu, Seok Bae Jang, Ken Samuel, Matthew Krause, Jon Phillips, Cathy H. Wu

**Affiliations:** 1 Georgetown University 37th and O Streets NW Washington DC 20057 USA; 2 The MITRE Corporation 7515 Colshire Drive McLean VA 22102 USA

## Abstract

Interest in information extraction from the biomedical literature is motivated by the need to speed up the creation of structured databases representing the latest scientific knowledge about specific objects, such as proteins and genes. This paper addresses the issue of a lack of standard definition of the problem of protein name tagging. We describe the lessons learned in developing a set of guidelines and present the first set of inter-coder results, viewed as an upper bound on system performance. Problems coders face include: (a) the ambiguity of names that can refer to either genes or proteins; (b) the difficulty of getting the exact extents of long protein names; and (c) the complexity of the guidelines. These problems have been addressed in two ways: (a) defining the tagging targets as protein named entities used in the literature to describe proteins or protein-associated or -related objects, such as domains, pathways, expression or genes, and (b) using two types of tags, protein tags and long-form tags, with the latter being used to optionally extend the boundaries of the protein tag when the name boundary is difficult to determine. Inter-coder consistency across three annotators on protein tags on 300 MEDLINE abstracts is 0.868 F-measure. The guidelines and annotated datasets, along with automatic tools, are available for research use.


**Comparative and Functional Genomics**
*Comp Funct Genom* 2005; **6**: 72–76.
Published online in Wiley InterScience (www.interscience.wiley.com). **DOI:** 10.1002/cfg.452

**Conference Paper**

### Protein name tagging guidelines: lessons learned

Inderjeet Mani[1]*, Zhangzhi Hu[1], Seok Bae Jang[1], Ken Samuel[2], Matthew Krause[1], Jon Phillips[1] and Cathy H. Wu[1]

[1] *Georgetown University, 37th and O Streets NW, Washington, DC 20057, USA*
[2] *The MITRE Corporation, 7515 Colshire Drive, McLean, VA 22102, USA*

*Correspondence to: Inderjeet Mani, Georgetown University, 37th and O Sts NW, Washington, DC 20057, USA. E-mail: im5@georgetown.edu*

Received: 9 December 2004
Accepted: 14 December 2004

### Abstract

**Interest in information extraction from the biomedical literature is motivated by the need to speed up the creation of structured databases representing the latest scientific knowledge about specific objects, such as proteins and genes. This paper addresses the issue of a lack of standard definition of the problem of protein name tagging. We describe the lessons learned in developing a set of guidelines and present the first set of inter-coder results, viewed as an upper bound on system performance. Problems coders face include: (a) the ambiguity of names that can refer to either genes or proteins; (b) the difficulty of getting the exact extents of long protein names; and (c) the complexity of the guidelines. These problems have been addressed in two ways: (a) defining the tagging targets as protein named entities used in the literature to describe proteins or protein-associated or -related objects, such as domains, pathways, expression or genes, and (b) using two types of tags, protein tags and long-form tags, with the latter being used to optionally extend the boundaries of the protein tag when the name boundary is difficult to determine. Inter-coder consistency across three annotators on protein tags on 300 MEDLINE abstracts is 0.868 F-measure. The guidelines and annotated datasets, along with automatic tools, are available for research use. Copyright © 2005 John Wiley & Sons, Ltd.**

**Keywords: nomenclature; protein names; guidelines; database curation; named entity tagging; inter-coder reliability**

### Introduction

With the enormous quantity and variety of high-throughput data being generated in the post-genome era, one of the major challenges in managing biological knowledge is to provide timely, accurate and consistent annotation of biological databases, such as primary DNA (GenBank) and protein sequence databases (UniProt) and many other secondary databases. Of particular value is annotation derived from experimentally verified data published in the scientific literature. However, the amount of such literature-based and manually-curated annotation is rather limited, due to the laborious nature of knowledge extraction from the literature. Interest in information extraction from the biomedical literature is motivated by the need to speed up the creation of structured databases representing the latest scientific knowledge about specific objects, such as proteins and genes. This has resulted in natural language processing technologies being utilized for biological literature mining and information extraction (Hirschman *et al.*, 2002).

We discuss here our experience in developing resources for one particular problem area, that of extracting protein names from MEDLINE abstracts. This task is fundamental to several other biological literature mining tasks, including the development of protein name ontologies and extraction of protein annotations (such as function and protein–protein interaction) from the literature.

Copyright © 2005 John Wiley & Sons, Ltd.

### The problem

Protein names show considerable variation because of the existence of multiple naming conventions. Researchers may name a newly discovered protein based on its function, sequence features, gene name, cellular location, molecular weight or other properties, as well as abbreviations and acronyms. For example, the EphB2 receptor, a protein involved in signalling in the brain, was initially referred to as 'Cek5', 'Nuk', 'Erk', 'Qek5', 'Tyro6', 'Sek3', 'Hek5', and 'Drt' before being standardized as 'EphB2' (Editorial, 1999). Potential standardization based on publishing guidelines and community consensus on naming are hard to enforce uniformly. Moreover, there are proteins whose status is tentative, and there is of course also a vast amount of legacy data.

Unfortunately, the previous research in protein and gene name tagging has been hampered in several ways. Some systems distinguish between protein and gene names while others do not, but the criteria for specifying when a protein or gene name should be tagged are not discussed. Thus, in addition to the lack of common datasets, it becomes very difficult to compare systems if one is unsure whether they are addressing the same problem. By using common *tagging guidelines*, it becomes possible for groups to share tagged data, compare automatic tagging results, and in general advance the field of biological information extraction. Also, *inter-coder reliability* is hardly ever reported (a notable exception is Hatzivassiloglou *et al.*, 2001). As a result, one has no real sense of the replicability and difficulty of the task is and how well the machine is faring relative to the upper bound of human performance.

The BioCreAtIvE evaluation is motivated by similar concerns, and is a very positive step that should address some of these issues. We believe that our resources and approach can be leveraged in such evaluations.

### Tagging guidelines v1

### Focus

Our first set of guidelines was relatively ambitious. We began with the assumption that it was crucial to annotate references to protein objects (including protein complexes and sets of protein objects), rather than simply annotating the protein names. References to genes, gene promoters, mutant genotypes, etc., were therefore not tagged. Thus, 'HypA' was tagged as a protein, while 'hypA', which refers to a gene, was not. *Ambiguity* between genes, proteins and genotypic strains was addressed by specific conventions.

### Tag type

We defined three tag types: (a) ⟨*protein*⟩ as a generic tag for most protein objects, including protein complexes (e.g. 'pyruvate dehydrogenase complex'); (b) ⟨*acronym*⟩ to tag acronyms or abbreviations; (c) ⟨*array-protein*⟩ to tag a list of proteins as a whole (e.g. 'FGF-1, -2, -4, -5, and -7').

### Tag extent

Our rules for tag extent were reasonably complex. A name was assumed to be made up of a pre-modifier chunk, a head, and a post-modifier chunk. Protein names were not tagged when used as modifiers for non-protein entities (e.g. 'elastase I promoter'). When the post-modifier in a name expressed a 'part-of' relationship (subunits or chains of a complex), the name was tagged as a whole, e.g. ⟨*protein*⟩ *subunit of NADH dehydrogenase (complex I)*⟨*/protein*⟩. However, if the part referred to a subregion of a protein or a polypeptide, such as 'c-terminal tail of the hLHR', only the head 'hLHR' was tagged. Other rules were defined for 'kind-of' and 'member-of' relations, as well as various other cases.

### Data and annotation procedure

We created two sets of 300 abstracts (called ABS1 and ABS2), each corresponding to 300 PIR (Wu *et al.*, 2003) protein entries that were randomly picked from about 5000 entries with curated information from high-quality underlying databases, such as Protein Sequence Database (PSD), *Saccharomyces* Genome Database (SGD) and LocusLink.

ABS1 was tagged by hand by one coder, using MITRE's Alembic Workbench (Day *et al.*, 1997). The human coder tagged nearly 3300 protein names in them. This experience provided a basis for developing a formal set of guidelines. ABS2 was then tagged according to the guidelines by three

Copyright © 2005 John Wiley & Sons, Ltd.

human coders using the Workbench. A1 was a co-author of this paper, while the others were biologists otherwise unconnected with this project.

### Assessment of v1

The inter-coder reliability metrics computed by a MUC-class named entity scorer used in the DARPA TIDES program is shown in Table 1. The scorer is strict, in that a candidate name and a reference name match (such a match is labelled 'Correct' in our tables) if and only if their respective text extents have exactly the same characters at exactly the same positions in the text.

Kappa ($\kappa\beta$) is often used to measure inter-coder reliability on classification tasks, but its extension to named entity extent is less clear. We consider each word position in the abstract, and compare whether or not the word at that position is a component of a protein name across coders. In addition to ignoring the boundary between contiguous protein names, this measure is generous and could give artificially high scores, because most words are not components of protein names, although chance agreement can be high. A related measure is used in Marcu *et al.* (1999) for computing $\kappa$ on discourse spans. This method gives $\kappa = 0.80$.

The ambitious ***focus*** on protein objects was a major reason for disagreement. Many of the cases of disagreement involved *ambiguity* of names that could refer to either genes or proteins. Moreover, many context-specific protein objects were tagged by some coders, even when they were very generic (e.g. protein, enzyme) or meaningless when taken out of context (e.g. 'E1 alpha').

We next consider *extent*. While the maximum protein name length was 12 words, about 93% of the tags were three words or less, and 86% of the tags were two words or less, and agreement on these was much higher. Coders were inconsistent in annotating pre- and post-modifiers and morphological affixes at the boundary of a name, and also in incorporating trailing punctuation in the tag. Nearly half such tags were off by just one word.

Finally, consider *tag types*. Acronym tagging achieved a 0.85 F-measure, but here the guidelines were not consistently followed. The array-protein tags were very hard to annotate (0.15 F-measure). This was because they were not clearly defined in the guidelines, e.g. a list of protein objects may or may not share a common core term.

Finally, coders showed *fatigue*, and often missed tagging multiple occurrences of the same protein name.

**Table 1.** Inter-coder reliability on protein tags (v1)

| Coders | Correct | Precision | Recall | F-measure |
|---|---|---|---|---|
| A1–A2 | 3091 | 0.750 | 0.748 | 0.749 |
| A1–A3 | 2766 | 0.8250 | 0.669 | 0.739 |
| A3–A2 | 2474 | 0.6 | 0.738 | 0.662 |
| Average | | 0.67 | 0.771 | **0.716** |

### Tagging guidelines v2

The above sorts of considerations led us to revise the guidelines, as discussed next.

### Focus

In the previous guidelines, when a protein name was followed by a non-protein object (e.g. 'elastase I gene promoter'), the protein name (e.g. 'elastase I') was not tagged. This was because of the focus on protein objects. In the modified guidelines, we defined the tagging targets as protein named entities (full names, acronyms or other symbolic names) used in the literature to describe proteins, or protein-associated or -related objects, such as domains, pathways, expression or gene. Thus, in the new guidelines, we have *⟨protein⟩elastase I⟨/protein⟩ gene promoter*, etc.

### Tag types and extent

In the revised guidelines, we used only two types of tags: *⟨protein⟩* and *⟨long-form⟩*. The ⟨long-form⟩ tag is designed to optionally extend the boundaries of ⟨protein⟩ tag when the name boundary is difficult to determine, thereby improving inter-annotator consistency. The long-form is only used in two situations (more details are at our website):

1. Organism names preceding a protein name may or may not be part of the protein name, e.g. the species name is tagged as part of the protein name if the protein name contains an acronym abbreviating the species name, e.g. *⟨protein⟩human growth hormone (hGH)⟨/protein⟩*, but *⟨long-form⟩human ⟨protein⟩IGF-II⟨/protein⟩⟨/long-form⟩*.

Copyright © 2005 John Wiley & Sons, Ltd.

2. When several protein entities share common terms, there may be only one name entity that can be easily tagged. We tag such an entity as a protein, while the list of entities together are tagged as a long-form, e.g. *⟨long-form⟩⟨protein⟩CSN subunits 4⟨/protein⟩, 5, 6⟨/long-form⟩*.

### Assessment of v2

The results on inter-coder reliability using the revised guidelines are much better. We present results for F-measure in Table 2 with three coders on ABS2. Note that the coders A1 and A3 were also involved in v1. The corresponding $\kappa$ scores are shown in Table 3.

### Related work

Other work on inter-coder reliability comes from Hatzivassiloglou *et al.* (2001), who had three annotators manually classify 550 terms found in 15 full-text articles from PubMed as 'gene', 'protein', 'mRNA', 'ambiguous' or 'wrongly extracted'. They found 77.58% pairwise agreement and 69.27% three-way agreement.

**Table 2.** Inter-coder reliability: F-measure (v2)

| Coders | Correct | Precision | Recall | F-measure |
|---|---|---|---|---|
| *⟨protein⟩* | | | | |
| A1–A3 | 4497 | 0.874 | 0.852 | 0.863 |
| A1–A4 | 4769 | 0.884 | 0.904 | 0.894 |
| A3–A4 | 4476 | 0.830 | 0.870 | 0.849 |
| Average | | 0.862 | 0.875 | **0.868** |
| *⟨longform⟩* | | | | |
| A1–A3 | 172 | 0.720 | 0.599 | 0.654 |
| A1–A4 | 241 | 0.837 | 0.840 | 0.838 |
| A3–A4 | 175 | 0.608 | 0.732 | 0.664 |
| Average | | 0.721 | 0.723 | **0.718** |

**Table 3.** Inter-coder reliability: $\kappa$ (v2)

| Coders | $\kappa$ |
|---|---|
| *⟨protein⟩* | |
| A1–A3 | 0.899 |
| A1–A4 | 0.930 |
| A3–A4 | 0.892 |
| Three-way | **0.932** |
| *⟨long-form⟩* | |
| A1–A3 | 0.657 |
| A1–A4 | 0.819 |
| A3–A4 | 0.662 |
| Three-way | **0.766** |

We now compare our annotated corpus with the GENIA corpus vs. 3.0.2 (Kim *et al.* 2003). The latter is a 2000-abstract corpus of biological literature compiled from the MEDLINE database and tagged with a set of hierarchical semantic classes. The GENIA corpus is focused on biological reactions concerning transcription factors in human blood cells, with the MeSH terms 'human', 'blood cell' and 'transcription factor' used as criteria for selecting abstracts.

The corpus has clearly a different focus from ours. Our corpora were chosen from the curated PIR database entries, which are not biased towards any particular area of biology, thus providing greater diversity in protein names for a given sample size. In addition, of course, our focus is on tagging protein names, a fundamental problem in automatically extracting experimental information of proteins from literature to assist protein database annotations.

The GENIA ontology classes corresponding to our protein name entities are 'protein complex', 'individual protein molecule', 'subunit of protein molecule', and 'peptide' (here we exclude peptides, as only naturally occurring peptides map to protein name objects, not artificial synthetic peptides). Based on our mapping, both corpora have a similar percentage (about 22%) of distinct protein names.

### Resources

A dictionary of 691 000 protein names was compiled from PIR entries. A case-insensitive exact matching of longest matching entries achieved an F-measure of 0.412 (0.372 Precision, 0.462 Recall) on ABS2. When used for preprocessing before coding, we found the dictionary lookup helpful with *standardization* and *extent*. It should also help with the *fatigue* problem, and thus could considerably further improve inter-coder reliability.

We have also developed several automatic taggers (also available) based on machine learning, which currently perform at about 0.59 F-measure, tested on both ABS2 and the 2000-abstract GENIA corpus version 3.0.2, with the latter being based on a mapping of GENIA tags to ours.

These results compare with a 0.40 F-measure for KEX (Fukuda *et al.* 1998) on ABS2, and

Copyright © 2005 John Wiley & Sons, Ltd.

are comparable with other work on GENIA. In the Hatzivassiloglou *et al.* (2001) study, their best automatic taggers were at 7–14% below human performance.

Our guidelines and annotated data (600 abstracts in all) are available to the community, along with a general corpus study and more detailed results. Relevant information about these resources, as well as the broader research framework, can be found at the following websites: **pir.georgetown.edu/iprolink/** (Hu *et al.*, 2004); and **complingone.georgetown.edu/~prot/**.

### Acknowledgements

The research into developing the necessary resources for extracting protein names from MEDLINE abstracts was supported by the National Science Foundation (ITR-0205470).

### References

Day D, Aberdeen J, Hirschman L, *et al.* 1997. Mixed-initiative development of language processing systems. In *Proceedings of the Fifth Conference on Applied Natural Language Processing.* Washington Marriott Hotel, Washington, D.C. 348–355.

Fukuda K, Tsunoda T, Tamura A, Takagi T. 1998. Toward information extraction: identifying protein names from biological papers. In *Proc Pacific Sympos Biocomp* **3**: 705–716.

Hatzivassiloglou V, Duboue PA, Rzhetsky A. 2001. Disambiguating proteins, genes, and RNA in text: a machine learning approach. *Bioinformatics* **17**(suppl 1): S97–106.

Hirschman L, Park JC, Tsuji J, Wong L, Wu CH. 2002. Accomplishments and challenges in literature data mining for biology. *Bioinformat Rev* **18**(12): 1553–1561.

Hu Z, Mani I, Hermoso V, Liu H, Wu CH. 2004. iProLINK: an integrated protein resource for literature mining. *Comput Biol Chem* **28**: 409–416.

Kim JD, Ohta T, Tateisi Y, Tsujii J. 2003. GENIA corpus — semantically annotated corpus for bio-textmining. *Bioinformatics* **19**(suppl 1): i180–182.

Marcu D, Romera M, Amorrortu E. 1999. Experiments in constructing a corpus of discourse trees: problems, annotation choices, issues. In *Proceedings of the Workshop on Levels of Representation in Discourse*, Edinburgh, July 1999; 71–78.

Editorial. 1999. Wanted: a new order in protein nomenclature. *Nature* **401**(6572): 411.

Wu CH, Yeh L-S, Huang H, *et al.* 2003. The protein information resource. *Nucleic Acids Res* **31**: 345–347.

Copyright © 2005 John Wiley & Sons, Ltd. 
